# Evidence-based restructuring of health and social care

**DOI:** 10.1371/journal.pmed.1002426

**Published:** 2017-11-14

**Authors:** Aziz Sheikh

**Affiliations:** Usher Institute of Population Health Sciences and Informatics, The University of Edinburgh, Edinburgh, United Kingdom

## Abstract

In this Perspective, Aziz Sheikh discusses research to evaluate health policy changes in the provision of care, commenting on a study by James Lopez Bernal and colleagues that examined specialist-dominated hospital care versus community-based care in the United Kingdom.

Governments around the world are grappling with how to respond to the challenges resulting from the epidemiological transition. Of particular concern is the increasing number of people living—for several decades—with 1 or more non-communicable disorders. The policy focus is centred on moving care away from the expensive specialist-dominated hospital sector to more community-based longitudinal care. The United Kingdom’s 2012 Health and Social Care Act, which gave control in England for the commissioning of care to clinicians working through Clinical Commissioning Groups (CCGs), represents one of the most ambitious and costly policy experiments to date [1]. This had the aims of supporting local decision-making, promoting innovation, and focusing attention on public health measures which, it was anticipated, would result in reductions in the need for specialist outpatient appointments and hospitalisations. In this issue of *PLOS Medicine*, however, James Lopez Bernal and colleagues report the results of their study finding that these benefits were not realised and that the intervention may have been associated with increased referrals to specialists [2].

## The need to restructure care

There is now across the world increasing policy interest in the need to restructure health and social care such that it is better suited to the needs of people living with long-term conditions. Although the emergence of the specialist hospital sector was an appropriate response to cater to the large numbers of people affected by life-threatening infectious disease epidemics, the burden of disease now predominantly arises from noncommunicable disorders. The use of hospitals as the mainstay of care for people living with long-term conditions is inconvenient for patients and an inefficient use of public resources.

The policy focus is therefore on seeing whether patients can be better managed in community care contexts where they can receive longitudinal care in close proximity to where they live, with an emphasis on supported self-management and coordination of care, and with a greater focus on population-based preventive care than is possible in hospital-dominated health systems [3].

## Integrating health and social care

Bradley and Taylor’s investigations in *The American Health Care Paradox* threw into sharp relief the need to consider expenditure on both health and social care in order to understand the relationship between expenditure and health outcomes [4]. This analysis, which has been widely debated in health policy circles, has underscored the need to integrate health and social care budgets in order to maximise the potential for health gains; for example, modest investments in home adaptations and mobility aids may be the difference between an individual’s ability to manage independently and a prolonged hospital admission.

Although the need to integrate health and social care policy is now widely appreciated, achieving this has proven challenging. The United Kingdom’s 2012 Health and Social Care Act represents one of the most important policy experiments in this respect [1]. In essence, this has involved passing financial control of local National Health Service (NHS) budgets to general practitioners through CCGs who were charged with procuring services on behalf of their patients. The underpinning assumption was that needs assessment and provision of care are best managed by those who are locally grounded. The Act thus resulted in a major shift of control and resources from the hospital sector to those providing front-line care, but as demonstrated by Lopez Bernal et al., this did not translate into a reduction of hospitalisations and was associated with an increased number of specialist outpatient referrals [2].

## Challenges to and opportunities for evidence-based policymaking

Health is largely won or lost on the basis of major health policy decisions, but these are seldom evaluated [5]. The reasons are complex, including the time and costs of undertaking such evaluations and the distinct possibility that they may reveal inconvenient truths. Politicians, especially those operating in liberal democracies such as the United Kingdom, are vulnerable to the effects of adverse publicity associated with what are often perceived as ‘failed’ government initiatives. These political challenges are real and not easily overcome until such time as there is a cross-party, longer-term approach to restructuring care.

More promising is that in many contexts it is now possible to exploit routinely collected data, thereby greatly reducing the time and costs of evaluating major policy initiatives on the restructuring of health and social care. This is well illustrated by the Lopez Bernal et al. study, which will have been undertaken much more rapidly and at a fraction of the cost of generating primary data [2]. As the United Kingdom’s data assets continue to mature, in addition to major recent government investments to make routine data more liquid—by improving access to and the ability to link data—and developing data science capacity, it will become possible to answer an increasing array of health policy questions within rapid timeframes at minimal costs. There is thus now, at least in the United Kingdom, the opportunity for a step-change in our ability to move towards evidence-based policymaking. What remains is the political maturity to see the value in such evaluations and, where necessary, iterate the policy approach in the light of their findings.

## Abbreviations

CCGs: Clinical Commissioning Groups
NHS: National Health Service
